# *C*. *elegans* as a new tractable host to study infections by animal pathogenic oomycetes

**DOI:** 10.1371/journal.ppat.1009316

**Published:** 2021-03-04

**Authors:** Manish Grover, Michalis Barkoulas

**Affiliations:** Department of Life Sciences, Imperial College London, London, United Kingdom; Geisel School of Medicine at Dartmouth, UNITED STATES

## Oomycetes: Plant and animal pathogens in a fungal disguise

The oomycetes or “water moulds” are diverse eukaryotic microbes that are found in both aquatic and terrestrial habitats. Based on their absorptive mode of nutrition, filamentous growth, and formation of spores for reproduction, it was initially thought that they are related to fungi, hence their name meaning “egg fungus” in Greek [1]. However, molecular data placed oomycetes within the Stramenopiles, so more closely related to brown algae and diatoms rather than fungi [2]. The lack of septa in hyphae and the presence of a cellulose and β-glucan instead of a chitin-rich cell wall differentiate oomycetes from fungi, while their heterotrophic lifestyle sets them apart from other Stramenopiles, which are largely photosynthetic [2].

**Fig 1:**
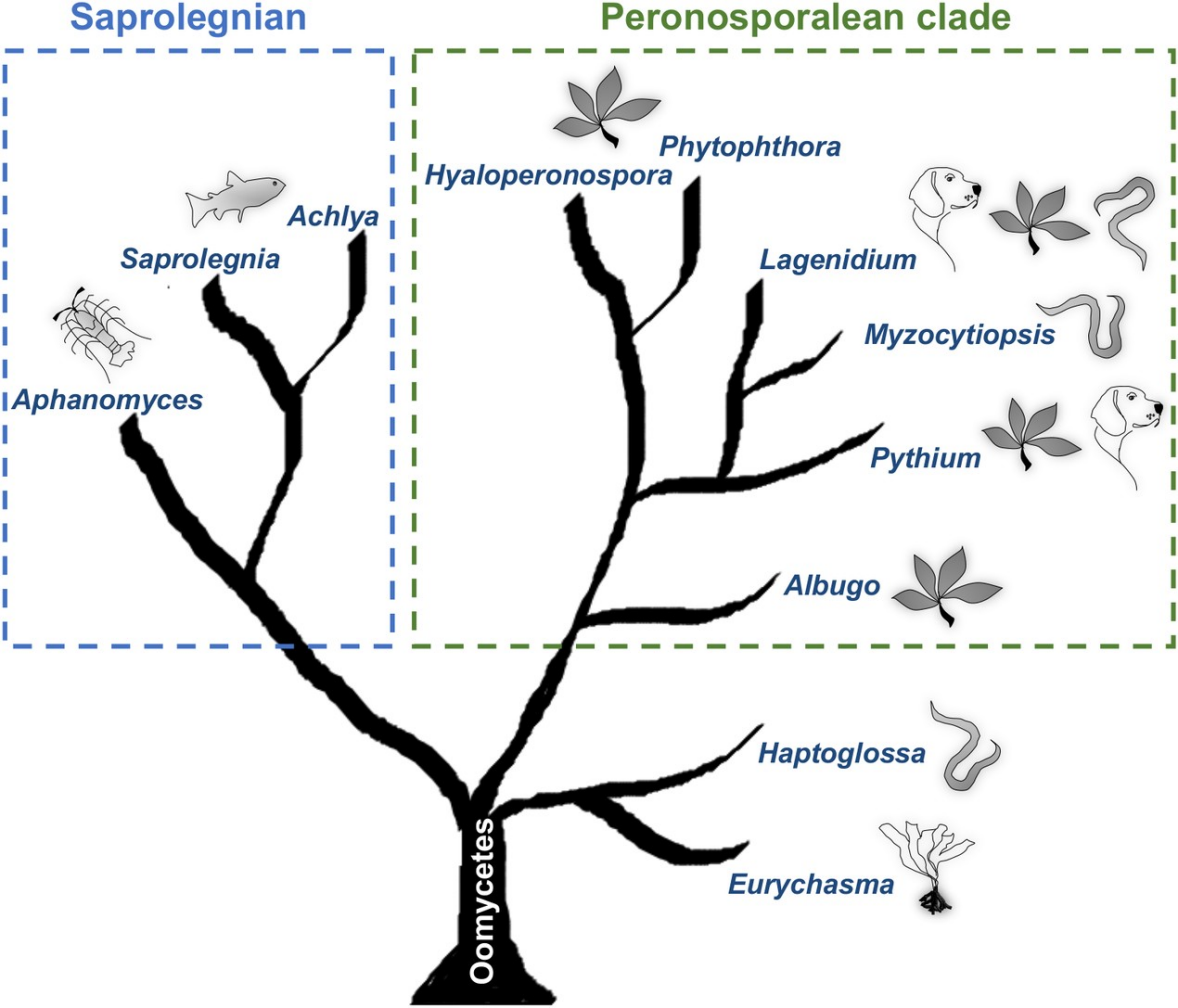
Cartoon representing the phylogeny of animal pathogenic oomycetes. The tree shows the phylogenetic position of nematode-infecting oomycetes in the context of the early diverging lineages (*Haptoglossa*/*Eurychasma*) and the 2 main oomycete clades (Saprolegnian and Peronosporalean), which all include animal pathogens. *Myzocytiopsis humicola* found to naturally infect *Caenorhabditis elegans* belongs to the Peronosporalean clade together with the plant pathogenic oomycete *Phytophthora infestans* and the mammalian pathogen *Pythium insidiosum*. The illustrations indicate the main host(s) infected by pathogen species within a certain genus, for example, nematodes in the case of *Haptoglossa*, brown algae for *Eurychasma*, and fish for *Saprolegnia*. Note that some genera include pathogens that infect both animal and plant hosts. The tree is adapted from [10] and modified to include nematode pathogens based on phylogenetic relationships described in [2,19].

While numerous saprophytic oomycetes live in aquatic and moist soil environments, it is the pathogenic oomycetes that have received the most attention. Most of our understanding about the oomycete biology comes from decades of research on the notorious plant pathogen *Phytophthora infestans*, which causes the late blight disease in potato and triggered the Great Irish famines in the mid-19th century. However, pathogenic oomycetes are not just restricted to plants and also represent an emerging threat to animal health (Fig 1). For example, members of the genus *Saprolegnia* and *Aphanomyces* infect fish and crustaceans, thus harm the aquaculture industry and threaten endangered species [3]. *Pythium insidiosum* infects a variety of mammals, such as horse, dog, cattle, and humans, leading to a disease known as pythiosis [4,5]. Although thought to be noncontagious, the clinical manifestations of pythiosis can progress into systemic fatal pathologies, and currently, apart from surgical removal of the infected tissue, there is no treatment available to cure this disease [6].

A constant coevolutionary battle shapes host–pathogen interactions. Pathogenic oomycetes use effectors as weapons to combat host responses and establish a successful infection [7]. For example, *P*. *infestans* displays a bipartite genome organisation having slow and fast-evolving regions, with the latter being enriched for genes encoding putative effectors that reside within repeats and transposable elements. Such plastic regions of the genome may allow oomycetes to rapidly evolve new virulence determinants and acquire species-specific means to overcome host resistance [8]. The repertoire of potential virulence factors that oomycetes use to infect animal hosts has also started to be understood [9–12]. Some virulence factors are shared between plant- and animal-infecting species, such as the extracellular lipid transfer proteins of the elicitin family or carbohydrate-binding module family 1 (CBM1) domain-containing proteins that both constitute pathogen-associated molecular patterns recognised by plant hosts [10,12,13,14]. However, effectors containing the well-studied N-terminal RxLR motif for translocation to plant cells are largely missing in *Saprolegnia parasitica* [10] or, when present, the motif is not required for translocation into fish cells, as in the case of a host-targeting effector that degrades RNA [11]. Instead, *S*. *parasitica* has an expanded repertoire of proteases [10]. In turn, hosts are able to sense pathogenic oomycetes to mount an appropriate defence response. For example, oomycete*-*derived cell wall carbohydrates have been shown to elicit an inflammatory response in fish cells [15]. Other immune responses include the phenoloxidase-mediated melanisation that correlates with oomycete resistance in crayfish [16] and the activation of Th2-type of cytokines in animal hosts infected with *P*. *insidiosum* [5].

## Oomycete infections in nematodes

In order to tackle the emerging oomycete threats, it is important to have the right models in place. Compared to plant pathogenic oomycetes, animal infections have been much more difficult to study in the lab due to paucity of tractable experimental systems. The discovery of a natural oomycete pathogen of the model nematode *Caenorhabditis elegans* [17] promises to bridge this gap.

**Fig 2:**
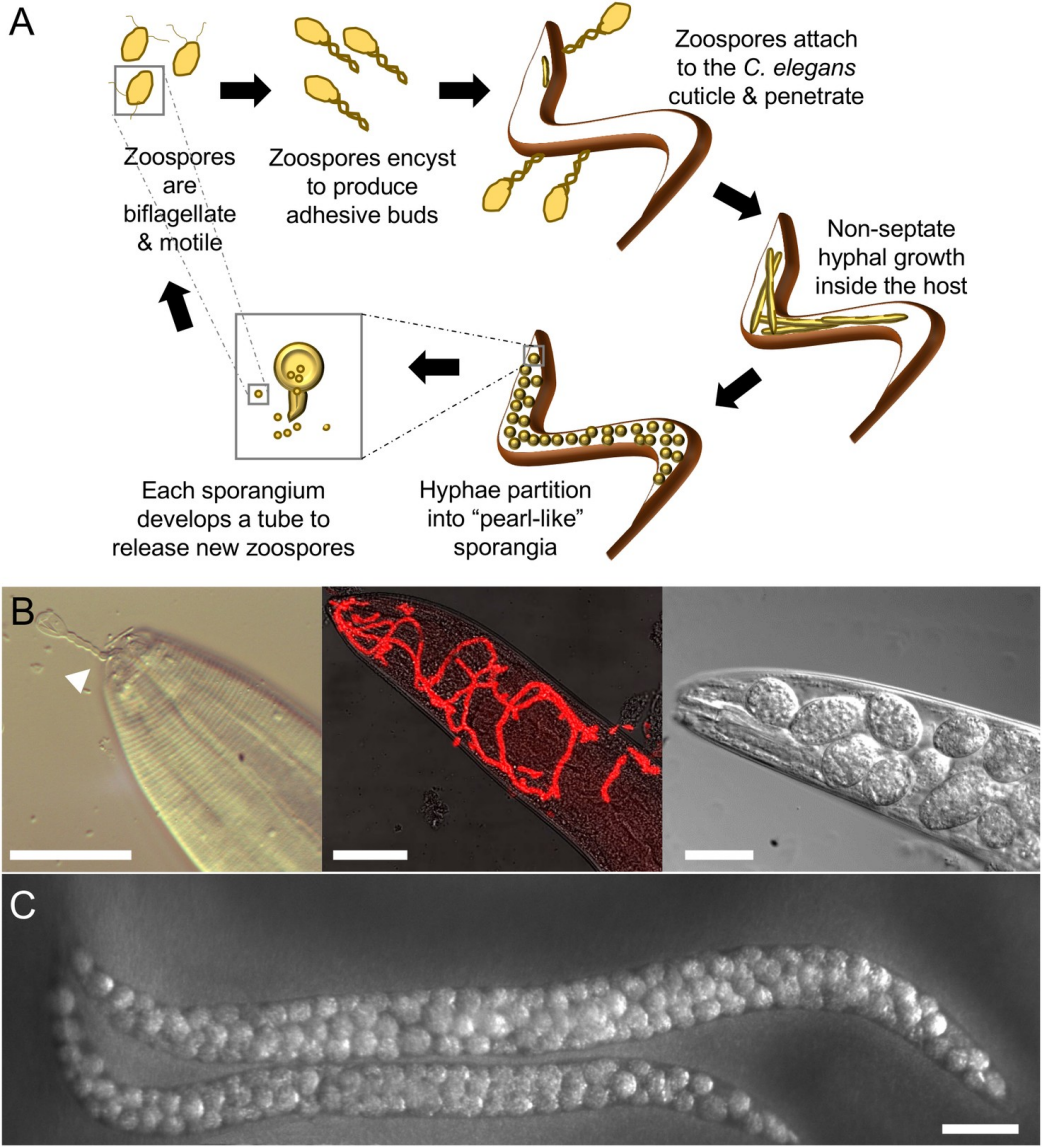
*Caenorhabditis elegans* infection by *Myzocytiopsis humicola*. (A) Cartoon describing the oomycete life cycle within nematodes. (B) Images show different stages of the infection process from pathogen attachment (left panel, arrowhead marks *M*. *humicola* adhesive bud attached to the nematode mouth) to early hyphal growth (middle panel, pathogen is visualised in red by 18S FISH), to development of “pearl-like” sporangia in the head region (right). (C) Late infection phenotype at the whole animal level with pathogen sporangia now developed throughout the body. Scale bars are 20 μm in B and 100 μm in C.

It all started with nematode sampling in Lisbon, which led to the recovery of a *C*. *elegans* isolate containing “pearl-like” structures in its entire body, suggestive of a putative infection (Fig 2). This pathogen was identified as the oomycete *Myzocytiopsis humicola* and has now been repeatedly found to be naturally associated with *C*. *elegans* [17]. Nematode infections by oomycetes are not unexpected; the first nematophagous oomycete was discovered by F.W. Zopf in the late 19th century, and several genera, such as *Chlamydomyzium*, *Gonimochaete*, *Haptoglossa*, and *Myzocytiopsis*, have been morphologically characterised as obligate parasites of nematodes [18,19]. However, it is the association of *M*. *humicola* with *C*. *elegans* that is particularly interesting, given the plethora of molecular and genetic tools that are available for research in this model organism.

The natural environment of *C*. *elegans* consists of soil, compost, rotting fruits, and leaves, where it goes through a boom-and-bust lifestyle based on food availability. In this habitat, *C*. *elegans* interacts with a plethora of microbes and pathogens, so it has evolved suitable means to respond to such biotic interactions [20]. In the absence of specialised immune cells and adaptive immunity, *C*. *elegans* relies on innate immune responses. Indeed, previous studies have identified evolutionarily conserved signalling pathways and species-specific immune effectors involved in the response to naturally occurring infections with bacteria [21], nodaviruses [22], microsporidia [23], and fungi [24], as well as lab-induced infections with human opportunistic pathogens such as *Pseudomonas aeruginosa* [25] and *Staphylococcus aureus* [26].

*M*. *humicola* infects through penetrating the collagenous cuticle of *C*. *elegans*, via attachment to specific regions, mostly the longitudinal ridges known as alae or near the mouth (Fig 2A and 2B). This contrasts with bacterial and fungal pathogens that commonly attach to the entire cuticular surface. Post-cuticle penetration, the oomycete spreads throughout the body cavity in the form of hyphae, without forming specialised sites of interaction with host cells, such as the haustorial interface observed during plant infections [27]. The pathogen hyphae absorb nutrients while degrading host tissues, swell, and partition into multiple pearl-like sporangia. The sporangia produce biflagellate zoospores, a characteristic of this group of pathogens, which are then released through exit tubes into the environment to form adhesive buds and infect new nematodes [17].

Nematode-infecting oomycetes occupy distinct phylogenetic positions (Fig 1), which may reflect diversity in the repertoire of virulence factors and infection strategies used. For example, members of the genus *Haptoglossa*, which represent a basal clade, employ specialised “gun cells” to shoot an infective sporidium into the body of nematodes [28]. Gun cells originate from encysted zoospores and contain an inverted tube that forms the sporidium, as well as a needle-like apparatus held under pressure by a large basal vacuole. The gun cells are kept ready to fire and upon contact with a nematode or pressure, the tube explosively everts, and the needle penetrates the cuticle leading to pathogen entry. It would be interesting to find out if *C*. *elegans* can also be infected by *Haptoglossa*. Together with the *M*. *humicola* model, this would allow a comparative analysis of infection and immunity mechanisms in evolutionary distinct oomycete lineages.

## Chitinase-like proteins as part of the host immune response

The *C*. *elegans* transcriptional response to *M*. *humicola* exposure is distinct to that against other nematode pathogens [17]. A hallmark of this response is the induction of chitinase-like (*chil*) genes in the epidermis, which modulate the host sensitivity to infection. Chitinase-like proteins (CLPs) are thought to cause modifications to the host cuticle, in a way that reduces pathogen attachment to initiate the infection, thereby providing host resistance [17]. This response strategy is reminiscent of callose deposition at the plant cell wall, which limits pathogen growth in response to oomycete detection [29]. CLPs are thought to arise from gene duplication of active chitinases, followed by mutations in their catalytic domain rendering them catalytically dead, although they can still bind chitin [30]. In other systems, CLPs have been studied in the context of helminth or bacterial infection [31,32], as well as inflammatory pathologies, such as asthma [33] and fibrosis [34]. In *C*. *elegans*, the *chil* gene family is expanded and includes at least 28 members compared to 4 in humans, so *chil* genes may exhibit functional redundancy or specificity for different pathogens. Pathogens have been shown to harbour their own CLPs, which can act to suppress host immunity [35].

Interestingly, the induction of CLPs was observed even upon exposure to an innocuous extract prepared from infected animals with *M*. *humicola* [36], so it is more likely to constitute a response to pathogen sensing rather than a response to pathogen-induced host perturbation. In addition, the *C*. *elegans* response to *M*. *humicola* was found to be modulated by chemosensory neurons, which through currently unknown mechanisms, trigger the induction of CLPs in the epidermis [36]. Pathogen recognition in *C*. *elegans* does not use classical innate immune receptors such as Toll-like receptors (TLRs), and the underlying mechanisms remain elusive. Therefore, the establishment of new tools, such as the *M*. *humicola* extract and *chil* gene induction as a binary readout, will facilitate efforts towards dissecting the host machinery involved in oomycete recognition.

## Future perspectives

We now have a tractable animal host to elucidate the role of immune response mediators, such as the CLPs and innate immunity pathways involved in detecting and fighting oomycete pathogens using molecular genetics. *C*. *elegans* can be used to identify systemic signals that coordinate the host response to infection at the whole organism level, such as those connecting sensory neurons to the responding epidermis upon pathogen recognition. To develop *M*. *humicola* as a pathogen model, a series of new tools will be required for genetic transformation, gene modification, and pure pathogen culture that are currently lacking. Genome and transcriptome analysis of the nematode-infecting species will start providing insights into potential virulence factors. Heterologous expression of pathogen virulence factors in *C*. *elegans* can be used to investigate their effect on host survival and physiology, while host-induced gene silencing may be useful to study their function during infection. Furthermore, attempts can also be made to test if nematode-killing oomycetes can serve as biocontrol agents for plant parasitic nematode infections damaging economically important crops [37]. The discovery of oomycetes as natural pathogens of *C*. *elegans* has opened up multiple new avenues for further research.
